# An exploration of smokeless tobacco product nucleic acids: a combined metagenome and metatranscriptome analysis

**DOI:** 10.1007/s00253-019-10232-3

**Published:** 2019-12-09

**Authors:** R. E. Tyx, A. J. Rivera, L. M. Keong, S. B. Stanfill

**Affiliations:** 1 Division of Laboratory Sciences, Centers for Disease Control and Prevention, Atlanta, GA USA; 2 Battelle Analytical Services, Atlanta, GA USA

**Keywords:** Tobacco, Smokeless, Microbiome, Metagenome, Metatranscriptome, Microbial communities, Metagenomics, 16S

## Abstract

Smokeless tobacco (ST) products are used worldwide and are a major public health concern. In addition to harmful chemicals found in these products, microbes found in ST products are believed to be responsible for generating harmful tobacco-specific nitrosamines (TSNAs), the most abundant carcinogens in ST. These microbes also contribute endotoxins and other pro-inflammatory components. A greater understanding of the microbial constituents in these products is sought in order to potentially link select design aspects or manufacturing processes to avoidable increases in harmful constituents. Previous studies looked primarily at bacterial constituents and had not differentiated between viable vs nonviable organisms, so in this study, we sought to use a dual metatranscriptomic and metagenomic analysis to see if differences exist. Using high-throughput sequencing, we observed that there were differences in taxonomic abundances between the metagenome and metatranscriptome, and in the metatranscriptome, we also observed an abundance of plant virus RNA not previously reported in DNA-only studies. We also found in the product tested, that there were no viable bacteria capable of metabolizing nitrate to nitrite. Therefore, the product tested would not be likely to increase TSNAs during shelf storage. We tested only a single product to date using the strategy presented here, but succeeded in demonstrating the value of using of these methods in tobacco products. These results present novel findings from the first combined metagenome and metatranscriptome of a commercial tobacco product.

**Electronic supplementary material:**

The online version of this article (10.1007/s00253-019-10232-3) contains supplementary material, which is available to authorized users.

## Introduction

Smokeless tobacco (ST) products are used by more than 300 million people worldwide, constituting a major public health concern globally (Agaku et al. 2014; NIH/CDC 2014; Wang et al. 2015). Besides toxicants and carcinogens designated by the International Agency for Research on Cancer (IARC), tobacco products also contain bacteria, fungi, and viruses (Tyx et al. 2016; Liu et al. 2013; Rivera et al., in submission). Certain microorganisms in tobacco contribute to the formation of mycotoxins, endotoxins, and nitrosamines; tobacco-specific *N*-nitrosamines (TSNAs) are thought to be the most abundant and potent carcinogens in ST products (Ayo-Yusuf and Connolly 2011; Fisher et al. 2012; Larsson et al. 2008; Lawler et al. 2013; Song et al. 2016; Zitomer et al. 2015). The presence of microbial populations also generate other potentially harmful constituents, such as endotoxins and other pro-inflammatory molecules (Rubinstein and Pedersen 2002; Tyx et al. 2016). There is a need for a deeper understanding of microbes that have an impact on the harmful chemicals found in ST products and which organisms remain viable in the purchased products. This information will provide a foundation for identifying means of mitigating the aforementioned negative impacts.

Microbial activity during the manufacturing of ST tobacco products and cigars contributes to the metabolism of reducing sugars that results in decreased harshness and improved flavor but also leads to the production of nitrite (Davis 1999). Tobacco fermentation is characterized by rapidly changing microbial community structures and consequently product chemistry. Cigar fermentation, the best characterized process to date, is characterized by a microbial succession and resulting chemical changes observed during an 18-day process (Di Giacomo et al. 2007). The dynamic metabolism can also result in production and accumulation of extracellular nitrite that reacts with tobacco alkaloids to form TSNA (Di Giacomo et al. 2007; Fisher et al. 2012). In a recent 16S community analysis, we predicted that respiratory (assimilatory) nitrate reductases could be involved in these processes when oxygen levels are low. These were predicted in abundance across products, encoded in the *nar* operon genes of *Staphylococcus*, *Corynebacterium*, and *Lactobacillus* genera, and certain members of the *Enterobacteriaceae* family (Tyx et al. 2016).

Most past investigations of microbial communities in ST products have used culture-independent methods, mainly targeting DNA marker sequences (16S, 18S, ITS) (Al-Hebshi et al. 2017; Han et al. 2016; Smyth et al. 2017; Tyx et al. 2016); these molecular approaches cannot differentiate DNA from living and that from deceased microorganisms. Because culture-independent experiments often rely on DNA isolations only, previous studies lacked the ability to differentiate live organisms from DNA persisting in the sample. One method to more accurately assess viable versus nonviable organism presence is metatranscriptomic analysis, which uses RNA to make a cDNA library that is then subjected to DNA sequencing. To date, only one RNA extraction from tobacco leaves has been previously described in the literature (Su et al. 2011). That particular study only focused on bacteria that could be washed off the leaves, and was not from a processed, ready-to-use product.

In the present study, we obtained a commercial moist snuff product bought from a tobacco wholesaler in the Atlanta area. A leading brand moist snuff was chosen as these type of products are the most popular of all ST sold in the USA (Richter et al. 2008). We characterized both RNA (as cDNA) and DNA libraries, in order to gain knowledge of the types of microbes, alive or otherwise, and their biochemical processes that may be active after production. The aim of this study was to evaluate a combined DNA and RNA shotgun sequencing approach to elucidate potentially viable microorganisms present in a moist snuff product and characterize genes being expressed by these microbes, especially those that are particularly active throughout processing (metagenome) or that are prevalent and likely viable in purchased products (metatranscriptome).

## Methods

### Tobacco samples

Tobacco samples were purchased locally through a third-party contractor to the US Centers for Disease Control and Prevention. Three tins of the product were combined in an amber glass bottle (250 ml) and homogenized by rotating. The product was kept under storage conditions at − 80 °C until DNA and RNA were extracted.

### Nucleic acid extraction

Nucleic acids were extracted from tobacco products using the MoBio PowerSoil Total RNA isolation kit (MO BIO Laboratories Inc.; Carlsbad, CA, USA) combined with the RNA PowerSoil DNA elution accessory kit (QIAGEN Inc.; Chatsworth, CA), with few modifications. Modifications included using the MPBio Lysing matrix E (MP Biomedicals, Santa Ana, CA, USA) in lieu of the bead-beating tubes from the PowerSoil kit, and the addition of a final cleanup step using QIAGEN DNEasy columns. RNA yield was quantified using a Qubit 2.0 with the RNA HS Assay (Thermo Fisher; Waltham, MA, USA).

### Library preparation and sequencing

Library preparation for the metagenome was performed using the TruSeq nano LT kit (Illumina, Inc.; San Diego, CA). The metatranscriptome library was prepared using NEBNext Ultra II RNA Library Prep Kit for Illumina (New England Biolabs; Ipswich, MA, USA). Library quality was assessed using an Agilent Bioanalyzer 2100 with a High Sensitivity DNA chip (Agilent Technologies; Santa Clara, CA, USA), and quantity was assessed using a Qubit 2.0 with the Qubit dsDNA HS Assay Kit (Thermo Fisher; Waltham, MA, USA). The metatranscriptome library was initially sequenced on an Illumina MiSeq using the MiSeq Reagent Nano Kit V2 (500 cycles) to provide a comprehensive assessment of library quality. Then the library was re-sequenced on a MiSeq Reagent Kit V2 (500 cycles) for greater sequencing depth.

### Data QC processing, filing, and annotation

All reads were subject to a QC protocol consisting of removal of adapter sequences, PhiX sequences, and quality cutoff of Q20 using SICKLE with minimum sequence length of 60 bp after quality truncation (SICKLE version 1.33) (Joshi and Fass 2011). Sequences were filed at NCBI SRA, accession SRR7719421. After QC, the metagenome sequencing run resulted in 12,626,111 paired reads (25,252,222 total) and the combined metatranscriptome data sequencing runs resulted in 13,564,027 paired reads (27,128,054 total). Metagenome assembly was performed using SPADES Meta v3.10.0 (Bankevich et al. 2012; Nurk et al. 2017) with default parameters, limited to 400-GB RAM and using 40 threads.

Prior to upload to IMG/M-ER and MG-RAST, paired reads from the metatranscriptome sequencing runs were combined using bbmerge.sh script with default settings, v8.92, https://sourceforge.net/projects/bbmap/, resulting in 97.1% of reads joining together into 13,174,617 sequences. The IMG Genome ID numbers are as follows: metatranscriptome: 3300012934, metagenome: 3300019856.

### 16S pipeline

Paired reads from two runs were catenated and the first 9 (sequencing run 1) or 10 bases (sequencing run 2) were removed from each sequence. Reads aligning to PhiX were then filtered and removed. Remaining reads were quality-filtered using SICKLE under default parameters (version 1.33). The paired reads were merged using USEARCH and also filtered using USEARCH with a “maxee” value of 1.0 (Edgar 2010). Merged, filtered reads were dereplicated using usearch “–fastx_uniques,” and operational taxonomic units (OTUs) were clustered using usearch “–cluster-otus” with “minsize” of 2, removing singletons (Edgar 2013). An OTU table was constructed using usearch “–usearch_global” command and taxonomy was assigned using usearch “–utax” algorithm with the RDP v15 trainset (from http://drive5.com/usearch/manual/utax_downloads.html), trained with the specified 250 utaxconfs file. OTUs with less than 99 % confidence to the 16S RDP trainset at the domain level were removed (66% of data), with the remaining 34.03% corresponding to 16S sequence (2,200,065/6,464,947 reads). The full 16S pipeline is in Supplemental Text A.

### Read mapping

BBMERGE (part of BBMAP utilities, https://sourceforge.net/projects/bbmap/) v36.02 was used to provide mapping coverage statistics.

### EMIRGE analysis

Paired read files were used with the EMIRGE script v0.60.3 (Miller 2013; Miller et al. 2011). A bowtie (Langmead et al. 2009) database was created from the Silva 111 SSU reference database (reference file name: SSURef_111_NR_tax_silva_trun.ge1200bp.le2000bp.fixed.sorted.97.fasta). EMIRGE output sequences were subjected to BLAST (megablast) search at NCBI against the NR/NT database (Altschul et al. 1990). The version of the script used was v0.60.3 with the parameters: –l 242 –i 208 –s 73 (metatranscriptome) and –l 242 –i 285 –s 69 (metagenome).

## Results

### Phylogeny and abundance approach

RNA was extracted from a leading brand ST product and converted to cDNA. Because numerous ways exist to compare phylogenetic abundance on shotgun metagenome data (metaphlan, phyloshop, megan, kraken, and R packages such as Phyloseq) (Huson et al. 2007; Mitra et al. 2011; Shah et al. 2011; Truong et al. 2015), phylogenetic data on the metagenome and metatranscriptome annotation were gathered using multiple methods. The first approach used results from files uploaded to the IMG/M-ER system, including assembled reads (metagenome) with corresponding read mapping average depth statistics, and for the metatranscriptome, uploading pair-joined reads. The second approach took advantage of the fact that without depleting ribosomal RNA first, most of the reads were ribosomal, mainly 16S and 23S. We used this knowledge to analyze the cDNA using a 16S community analysis pipeline; uparse was used for OTU picking, and utax was used for assigning taxonomy (Edgar 2010; Edgar 2013).

### Phylogenetic abundances and IMG/M-ER analysis of the metatranscriptome

Raw reads were filtered and processed for quality control, and read pairs were joined before uploading for annotation in IMG/M-ER. Of 13,174,617 reads uploaded to the IMG/M-ER system, 10,535,953 (80.0%) were annotated. Of those, 98.2%, or 10,239,347 of 10,535,953 total reads annotated in IMG/M-ER were attributed to RNA genes, mainly 23S and 16S rRNA, 64% and 34% of annotated reads, respectively. This level of rRNA is close to what should be expected from a sample without any ribosomal RNA clean up procedure (Rosenow et al. 2001). While the ribosomal sequences are not immediately useful in determination of genetic content, they are useful in defining taxonomic representation. More specifically, 3,550,824 (34.1%) of reads were identified as 16S in IMG/M-ER. This percentage agreed closely with the marker gene (16S) analysis of the overall data set (see “Methods”: 16S pipeline section)

In the IMG/M-ER system’s annotation of our metatranscriptome data, 1.58% of annotated reads (164,856) were attributed to protein-encoding genes. This relatively small number is likely not sufficient to give high confidence to low-expression genes in the metatranscriptome, but we felt it provided enough information to gain a high-level overview of what genetic content was being expressed and was even somewhat higher in number than the number of hits ascribed to protein-encoding genes in the assembled metagenome (21,628 annotated gene hits). In fact, the number of assigned genes with COG IDs, which we used here for analysis, was roughly double in the metatranscriptome, compared with the metagenome (31133 for the former, and 16350 for the latter).

Results for phylum-level abundance are found in Table 1. Bacteria were the most abundant in all annotated gene copies, 80.7% (84177/104279 classified gene copies at 30% + nucleotide identity) in the metatranscriptome, and 99.2% (4816480/4856474 gene copies at 30% + identity) in the metagenome. The four fungal phyla that IMG reported were *Ascomycota*, *Basidiomycota*, *Blastocladiomycota*, and *Chytridiomycota*. The metatranscriptome had very few sequences attributed to Fungi, 0.16% of gene copies (162/104279 gene copies). The metagenome reflected this lack of fungal sequences, as only 0.03% (1627/4856474) gene copies were attributed to these four phyla. Virus sequences were highly represented in the metatranscriptome at 18.8% of annotated gene copies (19576/104279 counts), but only 0.26% (13027/4856474 gene copies) in the metagenome. This was due to a high amount of RNA virus identified in the metatranscriptome sample, mainly attributed to *Virgaviridae*, the family of viruses that tobacco mosaic virus (TMV) belongs to.

**Table 1:** Abundance by domain and phylum (IMG/M-ER). Kingdom- and phylum-level abundance table using data from IMG/M-ER. This table uses output of sequences with at least 30% identity. Numbers represent relative abundances based on estimated gene copies, using average fold coverage per scaffold

Domain	Phylum	MG est. gene copies	MG %	MT est. gene copies	MT %
*Archaea*	*Crenarchaeota*	3	0.00	0	0.00
*Archaea*	*Euryarchaeota*	1587	0.03	21	0.02
*Archaea*	*Thaumarchaeota*	11	0.00	2	0.00
*Bacteria*	*Acidobacteria*	588	0.01	1	0.00
*Bacteria*	*Actinobacteria*	13227	0.27	1180	1.13
*Bacteria*	*Aquificae*	0	0.00	35	0.03
*Bacteria*	*Atribacteria*	1	0.00	0	0.00
*Bacteria*	*Bacteroidetes*	7791	0.16	671	0.64
*Bacteria*	*Balneolaeota*	0	0.00	5	0.00
*Bacteria*	*Candidatus Saccharibacteria*	4	0.00	0	0.00
*Bacteria*	*Chlamydiae*	281	0.01	16	0.02
*Bacteria*	*Chloroflexi*	265	0.01	2	0.00
*Bacteria*	*Cyanobacteria*	921	0.02	76	0.07
*Bacteria*	*Deinococcus-Thermus*	0	0.00	3	0.00
*Bacteria*	*Fibrobacteres*	0	0.00	2	0.00
*Bacteria*	*Firmicutes*	4766283	98.1	78811	75.6
*Bacteria*	*Fusobacteria*	5312	0.11	25	0.02
*Bacteria*	*Lentisphaerae*	0	0.00	2	0.00
*Bacteria*	*Marinimicrobia*	0	0.00	1	0.00
*Bacteria*	*Nitrospirae*	4	0.00	7	0.01
*Bacteria*	*Parcubacteria*	0	0.00	1	0.00
*Bacteria*	*Planctomycetes*	0	0.00	2	0.00
*Bacteria*	*Proteobacteria*	12548	0.26	3087	2.96
*Bacteria*	*Spirochaetes*	2165	0.04	16	0.02
*Bacteria*	*Synergistetes*	1414	0.03	3	0.00
*Bacteria*	*Tenericutes*	3207	0.07	182	0.17
*Bacteria*	*Thermodesulfobacteria*	0	0.00	2	0.00
*Bacteria*	*Thermotogae*	867	0.02	19	0.02
*Bacteria*	*Verrucomicrobia*	1	0.00	2	0.00
*Bacteria*	Unclassified	0	0.00	3	0.00
*Bacteria*		4816480	99.2	84177	80.7
*Eukaryota (Fungi)*	*Ascomycota*	1543	0.00	39	0.04
*Eukaryota (Fungi)*	*Basidiomycota*	76	0.00	4	0.00
*Eukaryota (Fungi)*	*Blastocladiomycota*	1	0.00	0	0.00
*Eukaryota (Fungi)*	*Chytridiomycota*	7	0.00	119	0.11
*Eukaryota (Fungi)*		1627	0.034	162	0.16
*Eukaryota*	*Annelida*	36	0.00	0	0.00
*Eukaryota*	*Apicomplexa*	20	0.00	4	0.00
*Eukaryota*	*Arthropoda*	66	0.00	3	0.00
*Eukaryota*	*Chlorophyta*	27	0.00	0	0.00
*Eukaryota*	*Chordata*	166	0.00	0	0.00
*Eukaryota*	*Cnidaria*	7	0.00	0	0.00
*Eukaryota*	*Nematoda*	1	0.00	0	0.00
*Eukaryota*	*Phaeophyceae*	1	0.00	0	0.00
*Eukaryota*	*Porifera*	16	0.00	0	0.00
*Eukaryota*	*Streptophyta*	24978	0.51	355	0.34
*Eukaryota*	Unclassified	22	0.00	2	0.00
Non-fungi *Eukaryota*		25340	0.52	364	0.35
*Viruses*	Retro-transcribing viruses	323	0.01	0	0.00
*Viruses*	dsDNA viruses, no RNA stage	12701	0.26	177	0.17
*Viruses*	dsRNA viruses	0	0.00	20	0.02
*Viruses*	ssDNA viruses	3	0.00	0	0.00
*Viruses*	ssRNA viruses	0	0.00	19379	18.58
*Viruses*		13027	0.27	19576	18.8
Totals		4856474		104279	

**Fig. 1:**
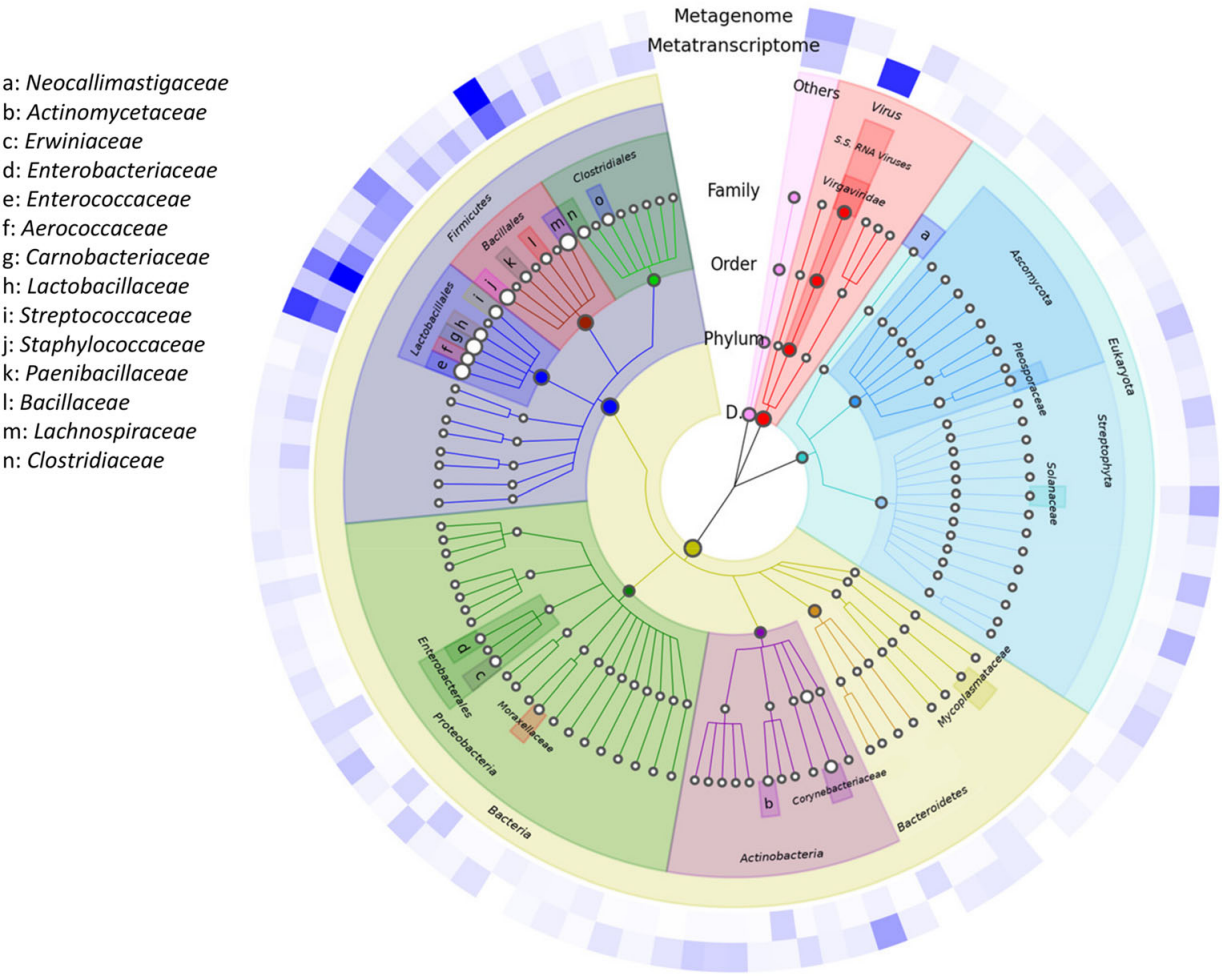
Cladogram representing taxonomic groups and relative abundance in the **a** metagenome and **b** the metatranscriptome of selected smokeless tobacco product. Raw counts were output from IMG/M system using Radial Tree. Data was processed by adjusting all abundances relative to a maximum relative abundance of 1, and then taking the square root of that number, to better illustrate lower abundance taxons. Twenty of the most abundant families are highlighted. Low-abundance phylogeny in either the metagenome or metatranscriptome was excluded and grouped into a category labeled “Others.” The “D.” in the center represents the domain level of taxonomy. GraPhlAn was used to create the cladogram (Asnicar et al. 2015)

**Fig. 2:**
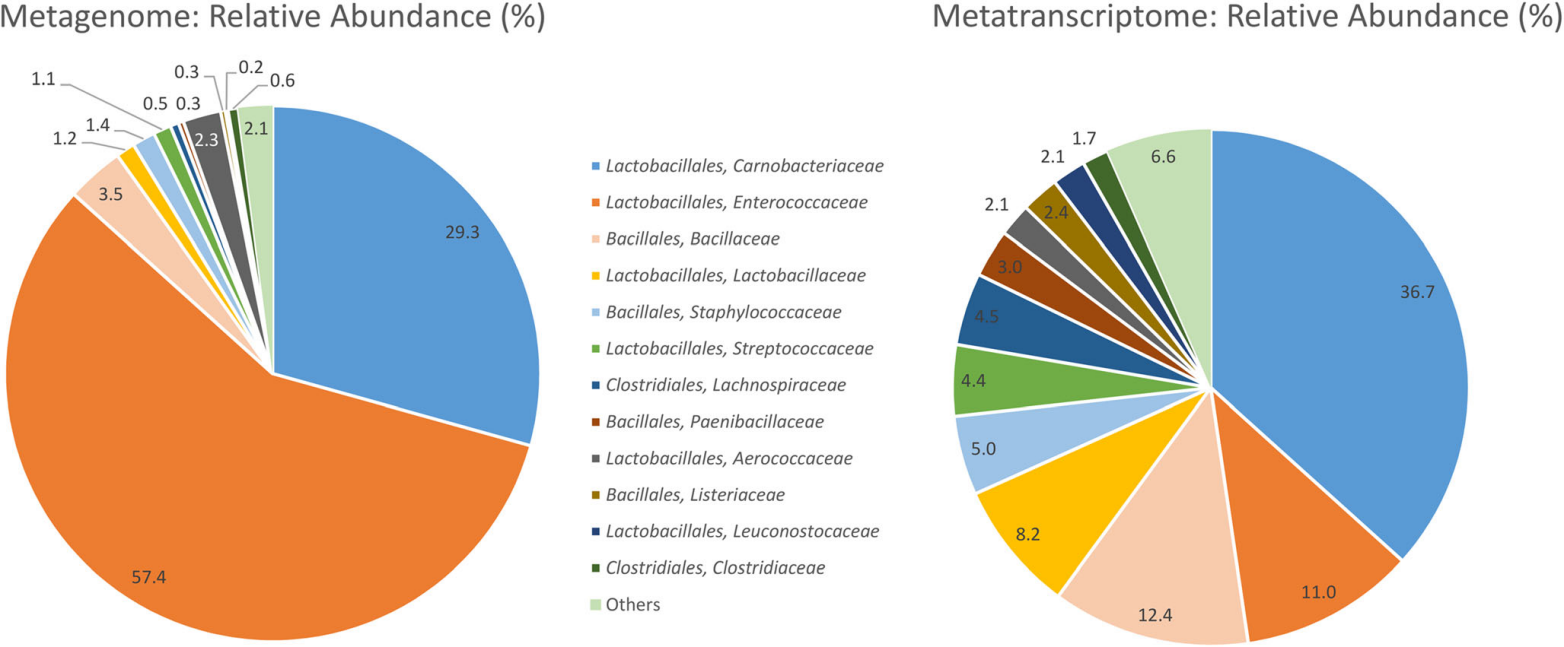
Distribution of the *Firmicutes* phylum families, highlighting the differences between metagenome and metatranscriptome. Using the data from the IMG/M-ER system (“Radial Tree” function), we constructed graphs highlighting the change in abundances between the metagenome and metatranscriptome for families present in the *Firmicutes* phylum

Overall, taxonomic abundance at the family level was determined using the “Radial Tree” command in IMG/M-ER and presented as Fig. 1, and in tabular form in Table 2. It should be noted that all low-abundance families, comprising of 111 families, were grouped into a category labeled “Others.” A square root transformation was used in Fig. 1 to create the figure in order to give better detail to lower abundance phylogeny. The relative abundance profile of the highly represented Firmicutes phylum is broken down further in Fig. 2. Tabulated data for Fig. 2 is given in Supplementary Table S1.

**Table 2:** Family-level identification of taxonomic groups found in the metatranscriptome of a leading brand smokeless tobacco product by identification in IMG/M-ER with at least 30% identity. The number represents the relative abundance based on estimated gene copies, which uses average fold coverage per scaffold. *MG*, metagenome; *MT*, metatranscriptome

Taxonomic description	Relative abundances (%)	
Phylogeny (family)	MG	MT
*Bacteria, Firmicutes, Lactobacillales, Enterococcaceae*	56	8.3
*Bacteria, Firmicutes, Lactobacillales, Carnobacteriaceae*	29	28
*Bacteria, Firmicutes, Bacillales, Bacillaceae*	3.4	9.4
Others	3.2	6.8
*Bacteria, Firmicutes, Lactobacillales, Aerococcaceae*	2.3	1.6
*Bacteria, Firmicutes, Bacillales, Staphylococcaceae*	1.4	3.8
*Bacteria, Firmicutes, Lactobacillales, Lactobacillaceae*	1.1	6.2
*Bacteria, Firmicutes, Lactobacillales, Streptococcaceae*	1.1	3.4
*Bacteria, Firmicutes, Clostridiales, Clostridiaceae*	0.6	1.3
*Bacteria, Firmicutes, Clostridiales, Lachnospiraceae*	0.5	3.4
*Bacteria, Firmicutes, Bacillales, Paenibacillaceae*	0.3	2.3
*Bacteria, Firmicutes, Bacillales, Listeriaceae*	0.3	1.8
*Bacteria, Firmicutes, Tissierellales, Peptoniphilaceae*	0.3	0.7
*Bacteria, Firmicutes, Bacillales, Planococcaceae*	0.2	0.5
*Bacteria, Firmicutes, Lactobacillales, Leuconostocaceae*	0.2	1.6
*Bacteria, Firmicutes, Erysipelotrichales, Erysipelotrichaceae*	0.2	0.6
*Bacteria, Firmicutes, Clostridiales, Ruminococcaceae*	0.1	0.6
*Bacteria, Proteobacteria, Pseudomonadales, Moraxellaceae*	0.0	0.9
*Bacteria, Actinobacteria, Actinomycetales, Actinomycetaceae*	0.0	0.8
*Viruses, SsRNA_viruses, Virgaviridae* (tobacco mosaic virus)	0.0	18

### Using 16S tools with shotgun sequencing data: cDNA 16S pipeline and EMIRGE results

Based on the large abundance of 16S sequence, we treated the data as a 16S microbiome data set and compared results with the IMG/M-ER abundance estimates. After removing OTUs that were likely not 16S (OTUs that had < 99% confidence at the domain level), 34% of sequences (385 OTUs) remained and were assigned taxonomy at the genus level (see cDNA 16S Results table, Supplementary Table S2). At the phylum level, 99.9% of all hits were *Firmicutes*, with only a small number attributed to *Proteobacteria* (0.004%, 92/2,200,065 reads in the OTU table). A total of 21 genera in 11 families were identified. It should be noted, however, that genus-level identifications in the *Carnobacteriacaeae* and *Enterococcaceae* families were mostly low confidence, with < 80% confidence at the family level of taxonomy (Supplementary Data File 1, OTU table). We hypothesized that there may be a related species or genus in the family that has not been identified previously. Supporting this data was the output from EMIRGE, an open-source software that attempts to assemble full-length 16S sequences from next-generation sequencing reads. Results of EMIRGE (Supplementary Table S3) generated as the top abundance sequence, a 16S sequence that is most closely related to the *Carnobacteriaceae* family, with a 94% identity at the genus level to the most similar genus, *Marinilactibacillus*. This sequence was found by nucleotide BLAST to be 99% identical to uncultured bacterium clone ncd537f06c1 (GenBank HM277344.1), a clone isolated from the popliteal fossa (kneepit) of a human. This appears to be the most dominant bacterium in the product as indicated by the highest abundance in the metatranscriptome (> 60% normalized relative abundance). As the *Marinilactibacillus* genus is fairly poorly characterized in IMG/M-ER, currently, with only three annotated genomes as of our submission date, many of the gene hits belonging this particular organism may not be assigned at genus or species level of taxonomy, and only assigned to family *Carnobacteriaceae*.

### Genetic content

**Fig. 3:**
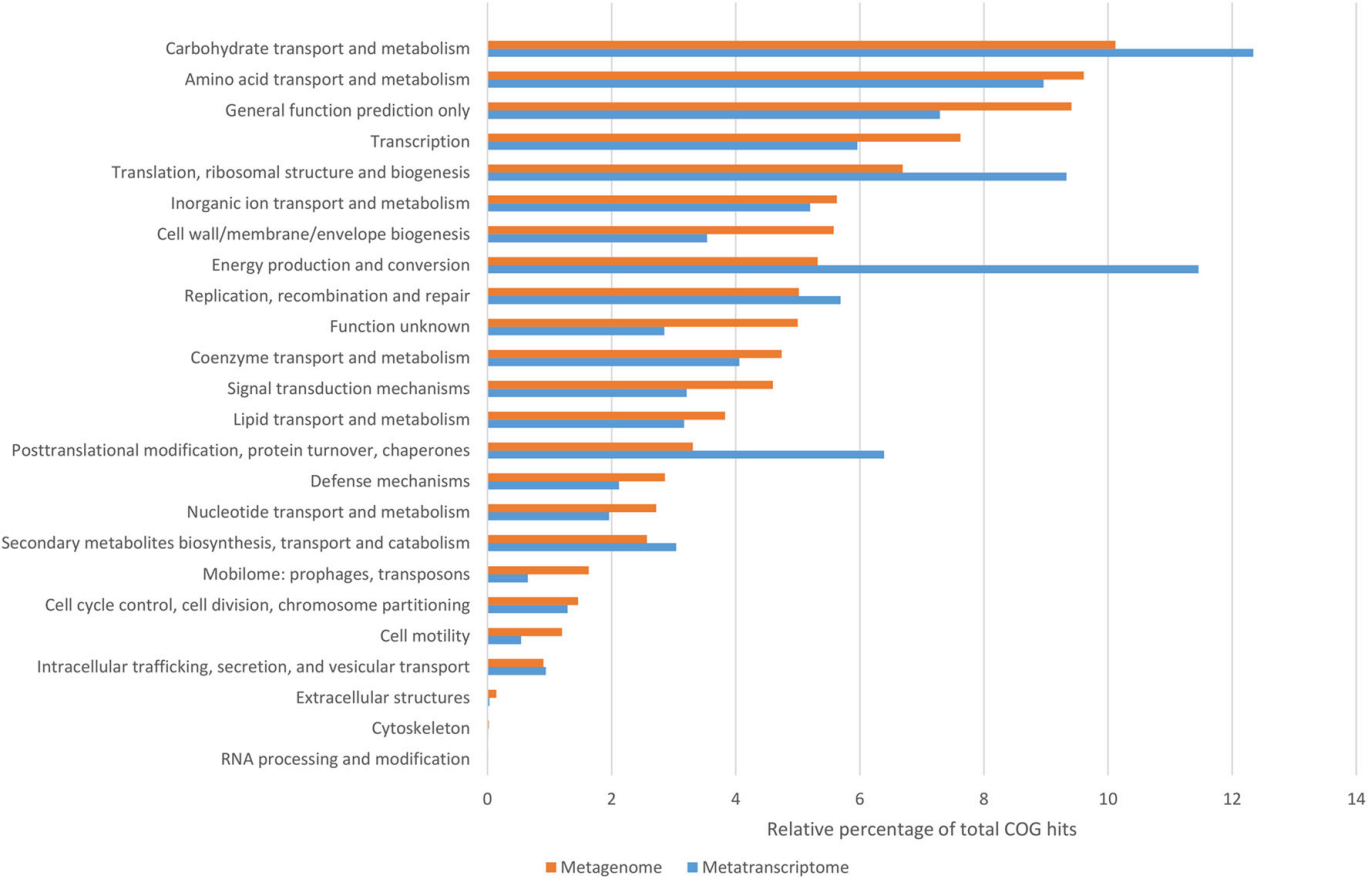
Graph of Functional Gene Content (as COGS) of metagenome vs metatranscriptome. Metagenome (orange bars) and metatranscriptome (blue bars) gene hits with COG functional annotation. These tables were combined from individual table outputs using the “with COG” link from “Metagenome Statistics” portion of the Genome Overview in IMG/M-ER. Relative percentages were from the “% of Total” column

**Fig. 4:**
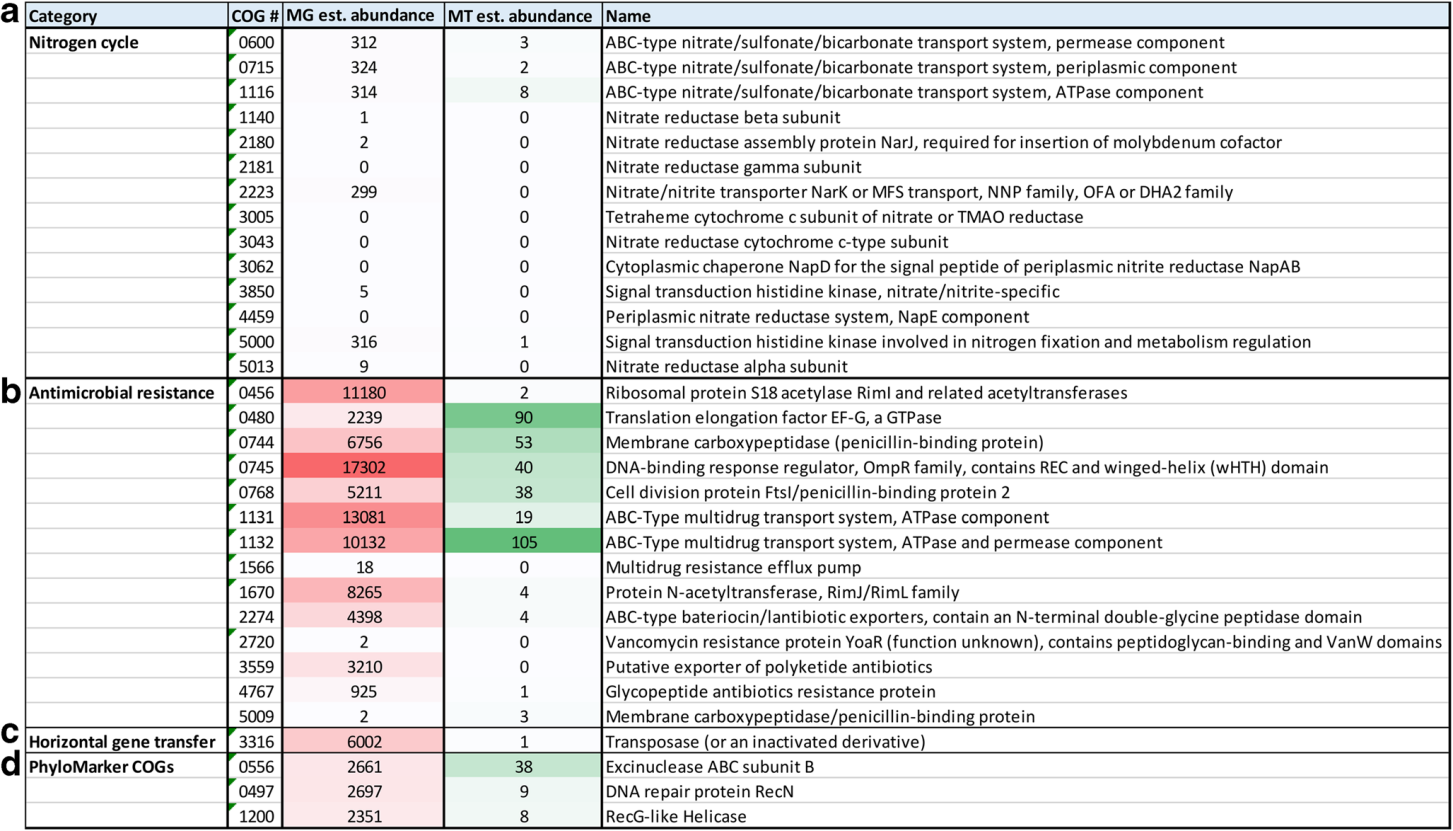
Targeted Categorical Gene Content heatmaps. Number of estimated gene copies of various COGS representing markers of **a** nitrogen cycle genes, **b** antimicrobial resistance genes, **c** gene transfer, and **d** phylogenetic marker COGS (as reference). Generated using “Functional Profile” tool in IMG/M-ER

Genetic areas of interest were explored using annotations for COGs (Clusters of Orthologous Groups). Figure 3 and Supplementary Table S4 display the metagenome and metatranscriptome abundance of various categories of gene function, represented by annotated genes in COGS. Several functional categories of interest are broken down in Fig. 4a–d. COGs for nitrogen metabolism (Fig. 4a), antimicrobial resistance (Fig. 4b), horizontal gene transfer (Fig. 4c), and phylogenetic markers (Fig. 4d) were investigated in both metagenome and metatranscriptome. Because we did not have ideal coverage of the transcriptome, it is likely that much of the lower expression transcript was missed. There was still enough coverage to be able to draw some conclusions from the data, however. A few classes of antimicrobial resistance–related genes were fairly abundant in the metagenome, but less were found in the metatranscriptome, and with some of the classes found in the metagenome almost or completely absent in the transcriptome (COG2274, COG3559, COG4767). Nitrogen metabolism genes (nitrate reductases, in particular) were identified in some abundance in the metagenome, but not in the metatranscriptome, except for a few ABC transport systems that are often promiscuous for other substrates or may have other functions. Antimicrobial resistance genes in these products were identified by COGS in IMG/M-ER and presented in the heatmap in Fig. 4b. Because many of these genes may represent normal nonresistant versions of structural molecules that can be resistant, little weight should be put on this data. Instead, a more detailed investigation using read mapping to a reference database (the Complete Antibiotic Resistance Database, CARD) for antimicrobial resistance genes was performed (Jia et al. 2017).

### Read mapping analysis: read mapping to metagenome and metatranscriptome

Specific targets of interest were investigated using read mapping. Reference sequences were obtained from NCBI (ncbi.nlm.nih.gov). In contrast to previously published tobacco metagenomes, tobacco vein clearing virus (TVCV) was not identified in high abundance in the DNA metagenome of this particular product (662/12626111 reads, 0.005%, Table 3, gene mapping: Metagenome) (Rivera et al., in submission).

**Table 3:** Results of read mapping of specific reference genes to metagenome. Specific genes were chosen to confirm presence and abundance of these genes in the raw sequence data

Species and strain	Gene symbol	Average fold coverage	% Reference bases covered	COG	min_align_ID %
*Bacillus pumilus* B4133	*narK*	7.86	100	COG2223	0.76
*Bacillus pumilus* B4133	*nirB*	8.33	98.5	COG1251	0.76
*Bacillus pumilus* B4133	*recG*	14.7	100	COG1200	0.76
*Tetragenococcus halophilus* DSM20339	*recG*	420	100	COG1200	0.76
Tobacco_vein_clearing_virus	TVCV	21. 7	100	N/A	0.76
*Nicotiana tabacum* 18S SILVA	18S	35.5	100	N/A	0.97
*Solanum tuberosum* 18S SILVA	18S	41.1	99.6	N/A	0.97

IMG/M-ER’s results suggested an abundance of plant RNA virus in the metatranscriptome, which we investigated using read mapping against a few plant viral genomes. Plant RNA viruses were detected at high levels in this product’s transcriptome, mainly TMV. TMV was identified in significant numbers in raw reads of the transcriptome and accounted for 0.2% of total reads (47809/2341452, Table 2), giving a 1528-fold average fold coverage of the TMV genome.

Reads from the cDNA library were mapped to the metagenome assembly. 99.2% of the metatranscriptome reads were mapped to the metagenome assembly using BBMAP’s default settings (76% minimum nucleotide identity). Mapping coverage and top results are listed in Table 4. Most of the contigs with the highest fold coverage in mapping contained at least a portion of a 16S or 23S gene.

**Table 4:** Read mapping of metatranscriptome to metagenome assembly, coverage of the 20 most abundant contigs, and presence of ribosomal or other genes of each particular contig

Contig no.	Average fold coverage	Contig length (b.p.)	Ribosomal-encoding or other feature
1794	199103	1114	23S
2333	98728	593	16S
3083	92077	297	16S
2393	46073	558	23S
3079	22112	299	16S
2502	19665	512	23S
2542	19497	502	16S
2590	151189	494	16S
1330	20659	2483	23S
757	19392	5969	16S
429	20183	10569	Contains gene(s) encoding cadmium/mercuric resistance
1630	121196	1460	Contains gene encoding type I restriction enzyme
650	24586	7163	Contains gene encoding type I restriction enzyme
1306	163311	2546	Contains gene encoding histidine kinase
794	46544	5696	23S
1150	107510	3299	Contains gene encoding transposase
1108	29666	3516	Contains gene encoding phage-related protein
949	43028	4537	Contains 6 annotated genes
710	52966	6418	Contains 8 annotated genes
402	20171	11122	Contains gene encoding RepA and contains 15 total annotated genes

### Read mapping to other databases (CARD, ICEBERG)

IMG/M-ER annotation suggests presence of numerous virulence factors including mobile genetic elements and antimicrobial resistance genes in the metagenome of this product. To obtain a more thorough knowledge of the presence of such genes, we conducted a read mapping analysis to known genes of interest using the CARD (v1_1_0, ref) and the Integrative and Conjugative Elements (ICEBERG, version 1) databases. Top results are displayed in Supplementary Table S5. No significant coverages were found for the metatranscriptome from these two databases. The most significant hit found in the metagenome to the CARD database was to the *dfrE* gene (for a dihydrofolate reductase) of *Enterococcus faecalis* (48-fold coverage of 92% of gene). Another gene was mapped at ~ 10-fold average coverage, identified as *cat86* of *Bacillus pumilus*. *FosB* of *Staphylococcus aureus* and *Erm*34 of *Bacillus clausii* (Bozdogan et al. 2004) were represented at 1-fold coverage; however, over only 66 and 67% of nucleotides of these genes were mapped, respectively. Also, most hits to the ICEBERG database should be considered “low confidence” because the “covered percentages” of all the top results failed to reach even 20%. With this in mind, read mapping of sequences to the ICEBERG database identified parts of two mobile elements which were highly represented in the metagenome with over 100-fold average coverage, to at least 10% of the whole ICEBERG sequences (which included multiple genes). The first element identified was a *Tn916*-like conjugative transposon, *Tn6079*, which carries multiple drug resistances, and the second element present was ICESsu_SC84_, an integrative conjugative element first identified in *Streptococcus suis* (Holden et al. 2009). Further analysis of the reads that mapped to Tn6079 indicated that these reads were nearly exclusively mapping to a sequence identified by BLAST as an insertion sequence (IS1216E), corresponding to a penicillin-resistant penicillin-binding protein gene (d-alanyl-d-alanine carboxypeptidase, *vanY*) found in *Enterococcus faecium* and *E. faecalis*. This element was identified as a transposon, Tn1546 (example sequence: GenBank KR047792.1). Reads that mapped to a portion of ICESsu_SC84_ were found to be mapping mainly to a small section that encoded a transposase, identified as ISSsu5, in *S. suis* and *E. faecalis* (example sequence: GenBank KX156278.1).

## Discussion

The present study used shotgun metatranscriptomic approach to confirm the presence of living or recently living microorganisms in ST products and confirmed previous reports that viable microbes are abundant in products (Smyth et al. 2017). We also found that techniques presented here, using an RNA expression profile (RNA-seq), are useful for observing the metabolic activities of microbes in smokeless tobacco products. Because these products yield only small quantities of extractable RNA, we were unable to use ribosomal RNA (rRNA) depletion on the present sample; therefore, we had only a limited amount of mRNA sequence to work with (roughly 400,000 reads, ~ 3% of all sequences). Fortunately, we were able to analyze this amount of mRNA efficiently with the IMG/M-ER system that gave indications of the processes occurring in the product microbiota. Further refinement of the extraction methods including the use of larger amount of the products in the isolation procedure may allow for a more suitable amount of RNA to be isolated to use in an rRNA depletion strategy, allowing further analysis of the transcriptome. We suggest further research into this to be well justified, as it would allow a better understanding of metabolic processes underway in smokeless tobacco products. These methods would also be quite useful in characterizing the processing steps including aging and fermentation, where presumably most nitrate is reduced and most nitrosamines are formed.

The combined shotgun metagenomics and metatranscriptomic approach provided us a unique view of the microbial community that included all domains of life, allowing us, for example, to now see RNA viruses that were not revealed in previous studies of the tobacco product metagenomes. This turned out to be especially important in tobacco samples, because most viruses found in this niche are likely to be RNA viruses (as are most plant viruses), and these can only be identified through an RNA to cDNA sequencing approach, at present. Although using a ribosomal RNA depletion on the RNA pool first would be essential for a dedicated functional study, this approach may be less effective in the ability to identify and classify reads attributed to plant viruses.

We found the use of a 16S pipeline for creating a community profile to be quite revealing as well, even with the phylogenetic resolution not ideal for differentiation to the genus and species levels due to the small average read size of the transcriptome library. While we would not suggest to rely on this data by itself to fully describe the microbial community, we did find that it was confirmatory to the metagenome data, and to what IMG/M-ER reported the abundances to be. Furthermore, using our approach provided for all V-regions having sequencing coverage, instead of just one or two regions being covered, which can also lead to bias (Klindworth et al. 2013). This method is further likely to introduce less bias than a typical marker gene analysis, as there is little or no amplification as compared with a traditional marker gene (such as 16S) study.

The *Firmicutes*, represented by the Class *Bacilli*, were found to be, by far, the most abundant class of bacteria, or any microbe, in this product. At the order level of classification, we find both *Lactobacillales* and *Bacillales* well represented in both the metagenome and metatranscriptome. The *Carnobacteriaceae*, followed by the *Enterococcaceae* and then the *Bacillaceae* were the most abundant families, with *Carnobacteriaceae* being more abundant, relatively, in the metatranscriptome than the metagenome and the *Enterococcaceae* appearing higher in abundance in the metagenome than in the metatranscriptome. Because the *Carnobacteriaceae* appeared to stay at similar relative abundances in both the metagenome and metatranscriptome, the decrease in abundance of the *Enterococcacaeae* in the metatranscriptome appeared to potentially be responsible for the increased relative abundances of all others in the metatranscriptome. A comparison of this product with products analyzed in previous marker gene studies indicates some similarities and some differences. Previous studies have identified the most abundant families in moist snuff to be *Bacillaceae*, *Staphylococcaceae*, *Aerococcaceae*, *Paenibacillaceae*, *Enterococcaceae*, and *Carnobacteriacae* (Al-Hebshi et al. 2017; Smyth et al. 2017; Tyx et al. 2016). The most abundant of these families in this particular product was not the highest abundance in any previous studies. This could be for a number of reasons; it could reflect the particular manufacturing of this product, microbes added during fermentation, or even a different starting population due to tobacco differences prior to processing.

Another interesting finding was a lack of Fungi in the metatranscriptome, but not in the metagenome. This may be reflecting the findings of Di Giacomo et al. (2007) who found that Fungi played a role earlier on in the tobacco fermentation process in Toscano cigars, but not in latter periods of the fermentation cycle.

Presence of plant RNA virus in this smokeless tobacco product is unsurprising, but warrants concern, nonetheless. Plant RNA viruses have been found to be abundant in human feces, and indeed, animals have been found to propagate some plant viruses, including TMV (Balique et al. 2013; Zhang et al. 2006). Tobacco users often have increased levels of anti-TMV antibodies, although nonusers were also found to be positive for anti-TMV antibodies (Liu et al. 2013). Because this product is used in a specific location of the mouth repeatedly, it is likely that the presence of TMV is contributing to chronic oral inflammation. Because chronic inflammation often plays a role in oncogenesis (Grivennikov et al. 2010), reduction of these viral particles could make products somewhat less harmful.

The genetic content of the metagenome of this product largely reflected what is present in the predominant species, of the genera *Marinilactibacillus*, *Atopostipes*, and *Tetragenococcus*. These genera have a similar capability (or lack thereof) to metabolize nitrate; annotated species of these genera all lack dissimilatory nitrate reductases and do not contain nitrate/nitrite antiporters (i.e., ABC transporters with similarity to the product of *narK* of *Escherichia coli*). Nitrate reductase genes identified in the metagenome scaffolds came from lower abundance genera, including *Corynebacterium*, *Siccibacter*, and *Staphylococcus*. The lack of nitrate reduction capabilities in the predominant organisms in the metatranscriptome suggest that the microbes found to be still viable in this particular product are likely not responsible for generating nitrite and thus leading to nitrosamine formation. As community diversity varies with brand, analyzing other products or brands that have microbes with respiratory nitrate-reducing capacities (Smyth et al. 2017; Tyx et al. 2016) would be beneficial to discover if they are actively utilizing these genes in at least some on the shelf products.

An abundance of horizontal gene transfer mechanisms and antimicrobial resistance genes has been suggested previously in imputed metagenome data, so these gene categories were explored in detail in this product. A few transposon and transposase genes were identified (using the ICEBERG database) and found to be highly covered in the metagenome. Antimicrobial resistance genes in this product were identified, mainly those found in *Enterococcus* and *Bacillus* genera. This was not surprising, given the niche these microbes occupy, where competition with fungi may be considerable. Overall, there were few hits to both integrative and conjugative genetic element (CARD), and antimicrobial resistance (ICEBERG) databases. A lack of relevant hits in the metatranscriptome suggests that while horizontal gene transfer of antimicrobial resistance genes could happen in the earlier stages of production, it is not likely active in the final product, at least for this particular product.

In conclusion, we found this combined approach to be powerful for producing a detailed analysis of tobacco product microbiome activity. We found evidence for potentially pathogenic bacteria, antimicrobial resistance genes, horizontal gene transfer pathways, and an unexpected abundance of viral nucleic acids. The approach presented here could be most effectively used to characterize the communities and expression of nitrate-reducing bacteria and fungi in an approach targeting the steps in ST processing where the most nitrate is being reduced, likely during the curing, aging, and fermentation steps. Organisms identified as living (by presence of RNA, transcribed to cDNA and sequenced) in this particularly finished, commercial product lacked the canonical capabilities to convert nitrate to nitrite efficiently. A study looking at the communities early in the processing of these products could have a large regulatory impact in that it would reveal species directly responsible for reducing nitrate and generating nitrite that results in nitrosamine formation. This information could help inform regulatory authorities as to potential changes in manufacturing that could be taken as preventative measures.

The presence of active microbes in tobacco products has long been known by the tobacco industry. However, presence of bacterial components and viral particles known to be antigenic or immunomodulatory is a cause for concern and justifies the need for more research to support approaches for making tobacco products less harmful.

## Electronic supplementary material

ESM 1 (PDF 844 kb)

ESM 2 (XLSX 44 kb)
