# Supplementary material for: An exploration of smokeless tobacco product nucleic acids: a combined metagenome and metatranscriptome analysis
Source: Appl Microbiol Biotechnol. 2019 Dec 9;104(2):751–63. doi: 10.1007/s00253-019-10232-3 (PMC6943401; doi:10.1007/s00253-019-10232-3)
Supplement: Supplementary file 1 — (PDF 844 kb) [file 253_2019_10232_MOESM1_ESM.pdf]

Applied Microbiology and Biotechnology

An exploration of smokeless tobacco product nucleic acids: a combined metagenome and metatranscriptome analysis

Tyx, R.E.[1]*, Rivera, A.J.[1], Keong, L.M.[2], Stanfill, S.B.[1]

[1] Division of Laboratory Sciences at the Centers for Disease Control and Prevention, Atlanta, GA

[2] Battelle Analytical Services, Atlanta, GA

Contact: Robert Tyx rtyx@cdc.gov Telephone: 1-770-488-4848, Fax: 1-770-488-4009

Supplemental Figures, Tables, and Text

Supplemental Table S1. IMG/M-ER analysis of relative abundances of Firmicutes families in Metatranscriptome and Metagenome, generated using output from "Radial Tree" function in IMG/M-ER.

**Order, Family**	**Metagenome**	**Metatranscriptome**
Lactobacillales, Enterococcaceae	57.4	11.0
Lactobacillales, Carnobacteriaceae	29.3	36.7
Bacillales, Bacillaceae	3.47	12.4
Lactobacillales, Aerococcaceae	2.30	2.07
Others	2.06	6.56
Bacillales, Staphylococcaceae	1.39	4.96
Lactobacillales, Lactobacillaceae	1.15	8.21
Lactobacillales, Streptococcaceae	1.08	4.45
Clostridiales, Clostridiaceae	0.61	1.65
Clostridiales, Lachnospiraceae	0.51	4.54
Bacillales, Paenibacillaceae	0.31	3.00
Bacillales, Listeriaceae	0.27	2.38
Lactobacillales, Leuconostocaceae	0.18	2.12

Supplemental Table S2. 16S Results of cDNA shotgun metatranscriptome library processed through 16S (Uparse/Utax) pipeline, and glommed at Family level of taxonomy. Taxonomy was called using the reference database, RDP (v15 representative set) and presented here in relative abundances.

Family	hits	% relative abundance	Highest OTU confidence
Carnobacteriaceae	1657359	75.3	0.985
Enterococcaceae	475663	21.6	0.998
Bacillaceae	28845	1.31	0.751
Staphylococcaceae	24415	1.11	0.310
Lactobacillaceae	8097	0.37	0.393
Aerococcaceae	3138	0.14	0.338
Paenibacillaceae	1441	0.066	0.393
Streptococcaceae	469	0.021	0.338
Leuconostocaceae	369	0.017	0.054
Planococcaceae	177	0.0081	0.751
Hydrogenophilaceae	90	0.0041	0.476
Comamonadaceae	2	<0.001	0.906
Total	2200065	100	

Supplemental Table S3. EMIRGE Results of 16S reconstructions

Metagenome					BLAST Results
>0	HM277344.1.1370	Prior=0.577926	Length=1370	NormPrior=0.603905	Uncultured bacterium clone ncd537f06c1 94%ID to *Marinilactibacillus*
>5	HQ716397.1.1518	Prior=0.154512	Length=1518	NormPrior=0.145716	Uncultured_bacterium 95%ID to *Atopostipes*
>1	AP012046.2445196.2446757	Prior=0.132162	Length=1558	NormPrior=0.121438	*Tetragenococcus halophilus*
>2	D88824.1.1512	Prior=0.119297	Length=1512	NormPrior=0.112952	*Tetragenococcus muriaticus* or *osmophilus*
>39	GU001891.1.1508	Prior=0.012747	Length=1430	NormPrior=0.012761	*Bacillus pumilus*
>70	HQ664563.15508.16998	Prior=0.003355	Length=1488	NormPrior=0.003228	Chloroplast
Metatranscriptome					
>0	HM277344.1.1370	Prior=0.406102	Length=1370	NormPrior=0.420824	Uncultured bacterium clone ncd537f06c1 94%ID to *Marinilactibacillus*
>11	GQ416919.1.1475	Prior=0.112984	Length=1475	NormPrior=0.108745	Uncultured *Atopostipes* or *Carnobacteriaceae*
>216	EU723856.1.1521	Prior=0.105142	Length=1510	NormPrior=0.098852	94%ID to *Tetragenococcus halophilus*
>33	EF517948.1.1687	Prior=0.092476	Length=1573	NormPrior=0.083461	Either *Lactobacillus* or Uncultured related to *Marinilactibacillus*
>4	AP012046.2445196.2446757	Prior=0.075289	Length=1543	NormPrior=0.069271	*Tetragenococcus halophilus*
>9	HQ716397.1.1518	Prior=0.055534	Length=1500	NormPrior=0.052560	95%ID to *Atopostipes*
>794	FR750972.1.1286_m12	Prior=0.047021	Length=1155	NormPrior=0.057796	Uncultured bacterium clone ncd537f06c1
>10	D88824.1.1512	Prior=0.033856	Length=1512	NormPrior=0.031788	*Tetragenococcus muriaticus*
>770	EF517948.1.1687_m02	Prior=0.031305	Length=1427	NormPrior=0.031144	Questionable maybe Uncultured bacterium similar to *Bacillus Staphylococcus* or *Tetragenococcus*
>247	AF349934.1.1456	Prior=0.011068	Length=1253	NormPrior=0.012540	*Tetragenococcus osmophilus* or *muriaticus*
>397	AB430339.1.1446	Prior=0.006255	Length=1440	NormPrior=0.006167	Uncultured bacterium clone ncd537f06c1
>243	FR750972.1.1286	Prior=0.002930	Length=978	NormPrior=0.004253	*Bacillus* sp. *pumilus* or *safensis*
>765	JF165770.1.1360_m01	Prior=0.002877	Length=1349	NormPrior=0.003028	Bactierum 94%ID to Uncultured clone ncd1931c03c1 related to *Marinilactibacillus*
>84	JF014539.1.1371	Prior=0.002860	Length=1370	NormPrior=0.002964	94%ID to Uncultured bacterium clone ncd537f06c1
>769	AF349934.1.1456_m02	Prior=0.002361	Length=1220	NormPrior=0.002747	93%ID to *Tetragenococcus halophilus*
>3	GQ280098.1.1445	Prior=0.002180	Length=1435	NormPrior=0.002157	*Bacillus* sp. *aerophilus xiamenensis* or *pumilus*
>40	GQ135642.1.1400	Prior=0.001950	Length=1169	NormPrior=0.002368	95%ID to *Marinilactibacillus*
>335	AM500811.1.1516	Prior=0.001771	Length=1392	NormPrior=0.001806	*Enterococcus* or *Vagococcus sp.*
>49	GQ135175.1.1353	Prior=0.001623	Length=730	NormPrior=0.003156	Uncultured *Carnobacteriaceae* possibly *Atopostipes*
>237	EU531780.1.1546	Prior=0.001460	Length=1513	NormPrior=0.001370	*Tetragenococcus muriaticus*
>70	AF286485.1.1531	Prior=0.001283	Length=1496	NormPrior=0.001218	*Bacillus* sp. *pumilus* or *safensis*
>307	GQ903406.1.1512	Prior=0.001015	Length=1505	NormPrior=0.000957	*Bacillus* sp. *pumilus* or *safensis*
>767	FR750972.1.1286_m01	Prior=0.000385	Length=1194	NormPrior=0.000458	96%ID to *Bacillus* sp.
>800	FR750972.1.1286_m15	Prior=0.000177	Length=1165	NormPrior=0.000216	97%ID to *Bacilllus* sp.
>824	FR750972.1.1286_m23	Prior=0.000079	Length=1194	NormPrior=0.000094	95%ID to *Bacillus* sp.
>225	EU589284.1.1482	Prior=0.000019	Length=447	NormPrior=0.000060	99%ID to *Bacilllus* sp.

Supplemental Table S4. Functional Gene Content by COG from IMG/M-ER. This table was from combined individual table outputs using the "with COG" link from "Metagenome Statistics" portion of the Genome Overview in IMG/M-ER.

	Metagenome		Metatranscriptome	
COG Categories	Gene count	% of total (29802)	Gene count	% of total (35627)
Amino acid transport and metabolism	2864	9.61	3192	8.96
Carbohydrate transport and metabolism	3016	10.1	4395	12.3
Cell cycle control, cell division, chromosome partitioning	434	1.46	459	1.29
Cell motility	358	1.20	194	0.54
Cell wall/membrane/envelope biogenesis	1664	5.58	1260	3.54
Chromatin structure and dynamics	4	0.01	1	0
Coenzyme transport and metabolism	1413	4.74	1446	4.06
Cytoskeleton	5	0.02	3	0.01
Defense mechanisms	853	2.86	755	2.12
Energy production and conversion	1584	5.32	4083	11.5
Extracellular structures	41	0.14	10	0.03
Function unknown	1491	5.00	1016	2.85
General function prediction only	2804	9.41	2596	7.29
Inorganic ion transport and metabolism	1677	5.63	1852	5.20
Intracellular trafficking, secretion, and vesicular transport	268	0.90	334	0.94
Lipid transport and metabolism	1142	3.83	1129	3.17
Mobilome: prophages, transposons	485	1.63	231	0.65
Nucleotide transport and metabolism	811	2.72	697	1.96
Posttranslational modification, protein turnover, chapero	986	3.31	2275	6.39
Replication, recombination and repair	1495	5.02	2028	5.69
RNA processing and modification	3	0.01	0	0
Secondary metabolites biosynthesis, transport and catabo	767	2.57	1082	3.04
Signal transduction mechanisms	1372	4.60	1143	3.21
Transcription	2271	7.62	2122	5.96
Translation, ribosomal structure and biogenesis	1994	6.69	3324	9.33
Not in COGs	38156		10373070	

Supplemental Table S5. CARD and ICEBERG top hit coverages from the metagenome read mapping to reference databases

ICEBERG Database ID	Avg fold	Length	Ref GC	Covered percent	Covered bases	Reads mapped
ICEberg|329|Tn6079|GenBank|GU951538|462..28872 Uncultured bacterium MID12 genomic sequence.	111	28411	0.3824	12.6852	3604	12031

ICEberg|234|ICESsu(SC84)|GenBank|FM252031|872762..961926 *Streptococcus suis* SC84 complete genome, strain SC84.	101	89165	0.3684	11.0144	9821	37348
ICEberg|377|ICESa2603|GenBank|AE009948|1256680..1311028 *Streptococcus agalactiae* 2603V/R, complete genome.	18.4	54349	0.3835	9.7334	5290	4453
ICEberg|76|ICESde3396|GenBank|EU142041|1..63668 *Streptococcus dysgalactiae subsp. equisimilis* strain NS3396 integrative conjugative element ICESde3396, complete sequence.	15.9	63668	0.3814	13.4981	8594	4714
ICEberg|326|Tn6087|GenBank|HQ663849|1..21169 *Streptococcus oralis* strain F.MI.5 transposon Tn6087, complete sequence.	14.3	21169	0.3823	15.1779	3213	1461
ICEberg|394|Tn6098|GenBank|CP001834|2295682..2347036 *Lactococcus lactis subsp. lactis* KF147, complete genome.	8.380	51355	0.3499	18.5727	9538	2119
**CARD Database ID**						
gb|AF028811|0-462|ARO:3002875|dfrE [*Enterococcus faecalis*]	48.19	463	0.3909	91.5767	424	102
gb|K00544|0-663|ARO:3002672|cat86 *[Bacillus pumilus*]	9.709	663	0.3499	100	663	28

Supplemental Text A.
Bioinformatics 16S pipeline for metatranscriptomic data
Starting with raw reads called run1_F.fq, run1_R.fq, run2_F.fq, run2_R.fq

Trim adapter sequences and phiX reads using bbduk.sh
bbduk.sh in=run1_F.fq in2=run1_R.fq t=20 –Xmx50g ref=bbmap/resources/nextera.fa.gz,bbmap/resources/truseq.fa.gz out1=run1_F_filtered.fq out2=run1_R_filtered.fq ktrim=r hdist=1 k=28 mink=12 minlength=60
bbduk.sh in=run2_F.fq in2=run2_R.fq t=20 –Xmx50g ref=bbmap/resources/nextera.fa.gz,bbmap/resources/truseq.fa.gz out1=run2_F_filtered.fq out2=run2_R_filtered.fq ktrim=r hdist=1 k=28 mink=12 minlength=60

Filter PhiX sequences out using bbduk.sh
bbduk.sh in=run1_F_filtered.fq in2=run1_R_filtered.fq t=20 –Xmx50g ref=bbmap/resources/phix174_ill.ref.fa.gz out1=run1_F_trimfilt.fq out2=run1_R_trimfilt.fq stats=run1_phixremovalstats.txt hdist=1 k=31
bbduk.sh in=run2_F_filtered.fq in2=run2_R_filtered.fq t=20 –Xmx50g ref=bbmap/resources/phix174_ill.ref.fa.gz out1=run2_F_trimfilt.fq out2=run2_R_trimfilt.fq stats=run1_phixremovalstats.txt hdist=1 k=31

Trim 9 bases off 5' end (run1)
fastx_trimmer –i run1_F_trimfilt.fq –f 9 –o run1_F_trimfilt_9bpremoved.fq
fastx_trimmer –i run1_R_trimfilt.fq –f 9 –o run1_R_trimfilt_9bpremoved.fq
Trim 10 bases off 5' end (run2)
fastx_trimmer –i run2_F_trimfilt.fq –f 9 –o run2_F_trimfilt_9bpremoved.fq
fastx_trimmer –i run2_R_trimfilt.fq –f 9 –o run2_R_trimfilt_9bpremoved.fq

Quality filter using SICKLE 1.33
sickle -pe –f run1_F_trimfilt_9bpremoved.fq –r run1_R_trimfilt_9bpremoved.fq -o run1_F_trimfilt_9bpremoved_sickle.fq –p run1_R_trimfilt_9bpremoved_sickle.fq –t sanger –s run1_sickle_singles.fq

Catenate both runs into single forward and reverse read files
cat run1_reads_F.fq run2_reads_F.fq > catenated_reads_F.fq
cat run2_reads_R.fq run2_reads_R.fq> catenated_reads_R.fq

Merge reads
usearch9.1 –fastq_mergepairs catenated_reads_F.fq –relabel @ -fastqout merged_1.fq

Filter using Usearch v7.0.1090
usearch7 –fastq_filter merged_1.fq –fastq_maxee 1.0 –fastaout filtered_1.fa

split filtered file
head –n 6537796 filtered_1.fa > filtered_1_1.fa

Dereplicate
usearch9.1 –fastx_uniques filtered_1_1.fa –fastaout uniques_1.fa

Cluster sequences into OTUs
usearch9.1 –cluster_otus uniques.fa –minsize 2 –otus otus.fa –relabel Otu

Assign taxonomy using Utax
usearch9.1 –utax otus.fa –db rdp_v15_250ref.udb –strand both –utaxout utax_out_v91.txt

Make OTU table
usearch9.1 –usearch_global merged_1.fq –db otus.fa -otutabout otutab.txt –biomout otutab_1.biom

Add taxonomy to biom file
biom add-metadata –i otutab_1.biom –o otutab_t_tax.biom --observation-metadata-fp utax_out_v91.txt --observation-header OTUID,taxonomy --sc-separated taxonomy

Convert biom to .tsv file
biom convert –i otutab_1_tax.biom –o otutab_tax.txt --to-tsv --table-type="OTU table" --header-key taxonomy
